# OCT4 and PAX6 determine the dual function of SOX2 in human ESCs as a key pluripotent or neural factor

**DOI:** 10.1186/s13287-019-1228-7

**Published:** 2019-04-18

**Authors:** Shuchen Zhang, Emma Bell, Huihan Zhi, Sarah Brown, Siti A. M. Imran, Véronique Azuara, Wei Cui

**Affiliations:** 1 Institute of Reproductive and Developmental Biology, Department of Surgery and Cancer, Faculty of Medicine, Imperial College London, London, W12 0NN UK; 2 Beijing Advanced Innovation Center for Food Nutrition and Human Health, China Agricultural University, Beijing, 100193 China; 3 Present address: Princess Margaret Cancer Centre, University Health Network, Toronto, M5G1L7 Canada

**Keywords:** SOX2, OCT4, PAX6, Neural differentiation, Human embryonic stem cells

## Abstract

**Background:**

Sox2 is a well-established pluripotent transcription factor that plays an essential role in establishing and maintaining pluripotent stem cells (PSCs). It is also thought to be a linage specifier that governs PSC neural lineage specification upon their exiting the pluripotent state. However, the exact role of SOX2 in human PSCs was still not fully understood. In this study, we studied the role of SOX2 in human embryonic stem cells (hESCs) by gain- and loss-of-function approaches and explored the possible underlying mechanisms.

**Results:**

We demonstrate that knockdown of SOX2 induced hESC differentiation to endoderm-like cells, whereas overexpression of SOX2 in hESCs enhanced their pluripotency under self-renewing culture conditions but promoted their neural differentiation upon replacing the culture to non-self-renewal conditions. We show that this culture-dependent dual function of SOX2 was probably attributed to its interaction with different transcription factors predisposed by the culture environments. Whilst SOX2 interacts with OCT4 under self-renewal conditions, we found that, upon neural differentiation, PAX6, a key neural transcription factor, is upregulated and shows interaction with SOX2. The SOX2-PAX6 complex has different gene regulation pattern from that of SOX2-OCT4 complex.

**Conclusions:**

Our work provides direct evidence that SOX2 is necessarily required for hESC pluripotency; however, it can also function as a neural factor, depending on the environmental input. OCT4 and PAX6 might function as key SOX2-interacting partners that determine the function of SOX2 in hESCs.

**Electronic supplementary material:**

The online version of this article (10.1186/s13287-019-1228-7) contains supplementary material, which is available to authorized users.

## Background

Cell type-specific transcription factors (TFs) play essential roles in configuring cellular epigenetic landscapes, regulating gene expression and thus determining cell fates [1–3]. Sox2 is one such TF required for maintaining the pluripotency in embryonic stem cells (ESCs) and early embryonic cells and inducing pluripotency from somatic cells via reprogramming [4–6]. In addition to its function in pluripotency, Sox2 exhibits distinct expression dynamics upon ESC differentiation to neuroectoderm and mesendoderm (Additional file 1: Figure S1). It is highly expressed in the neuroectoderm whilst quickly downregulated in the mesendoderm [7–9]. Furthermore, Sox2 has an important role in maintaining neural progenitor properties and converting fibroblasts into neural stem cells [10, 11]. Therefore, it has been proposed that Sox2 is not only a vital pluripotency factor but also a neuroectodermal lineage specifier [8, 12].

Genetic manipulation technology provides us with a powerful tool to explore the function of Sox2. Mouse embryos with germ-line deletion of *Sox2* fail to generate pluripotent epiblast but successfully form trophectoderm [13]. Similarly, elimination of *Sox2* in mouse ESCs (mESCs) results in the loss of pluripotency, which is coupled with differentiation to trophectoderm-like cells [14]. However, overexpressing *Sox2* in mESCs was also shown to induce cell differentiation to a wide range of cell types in one report [15], whereas in another report, this had no clear effect on mESC self-renewal but promoted neuroectoderm differentiation upon release from self-renewal [16]. In hESCs, the results are differing. *SOX2* deficiency was shown to compromise pluripotency in some reports [17, 18], whilst, in the other, neither reduction nor overexpression of *SOX2* were shown to affect self-renewal [4]. Nonetheless, upon the release from self-renewal, high levels of *SOX2* enhance neuroectoderm differentiation [4]. With these discrepant results, further studies are required to investigate the mechanisms underlying the pleiotropic effects of Sox2 in ESCs, especially at the exit of pluripotency.

Sox2, like other Sox family transcription factors, requires a cooperating binding partner to bestow its transcriptional regulatory functions [19]. Thus, it is plausible that the functions of Sox2 are considerably influenced by its binding partners. It has been well established that Sox2 and Oct4 form heterodimer in both mouse and human ESCs, which cooperatively activate pluripotency-related genes, including themselves and *Nanog* [14, 20]. In mouse embryos and mESC neural differentiation, Sox2 is reported to work with Zic, Otx2 and Pou factors for the neural development and formation of neural progenitor cells (NPCs) [5, 21]. However, SOX2-interacting partners that assist SOX2 for neural specification in human remain obscure. PAX6 has been identified to be expressed very early during hESC neural differentiation and human embryonic brain development [7, 22]. It also plays a crucial role in hESC neural differentiation [22]. However, it is unclear whether these two factors have any connections.

In this study, by manipulating *SOX2* expression in hESCs, we show that substantially diminishing SOX2 leads to hESC differentiation to endoderm-like cells, whereas the outcome of overexpressing *SOX2* is affected by external cues, either to retain self-renewal pluripotency or to promote neural specification. Mechanistically, we revealed that this dual function of SOX2 is likely determined via dynamic exchange of its interacting TF partners from OCT4 for self-renewing hESCs to neural TFs, such as PAX6, for neural differentiation. SOX2-OCT4 and SOX2-PAX6 control distinct target gene sets and differentially regulate OCT4 and BRN2 expression. Together, our findings provide strong evidence for the dual function of SOX2 in hESCs and illuminate the possible molecular pathways by which SOX2 mediates its distinctive roles in hESCs.

## Methods

### Cell culture and differentiation

Human ESC lines H1 and H7, purchased from WiCell Research Institute (Madison, WI, http://www.wicell.org), were routinely cultured in Matrigel-coated plates with mouse embryonic fibroblast-conditioned KSR medium supplemented with 4–10 ng/ml of bFGF (MEF-CM) as described previously [23, 24]. Briefly, the hESCs were fed daily with the medium and propagated mechanically every 5–7 days after collagenase IV treatment in a 1:3 ratio. The cells are regularly screened to make sure no mycoplasma contamination. Neural differentiation of hESCs was performed in a neural differentiating medium as previously described [7, 25]. Upon formation of neural progenitor cells (NPCs), the NPCs were maintained in NPC culture medium and propagated with TrypLE (Thermo Fisher Scientific). Neurons and astrocytes were produced by withdrawing bFGF and EGF from N2B27 medium for 1–2 weeks.

### Construction of plasmids

SOX2 cDNA was initially amplified from a PCR reaction by using the OKSIM plasmid (Addgene #24603) [26] as a template and with the primers containing restriction enzyme sites, FseI and AscI in the forward and reverse primers, respectively (Additional file 1: Table S1). The FseI/AscI fragment of SOX2 PCR product was then inserted into pCS2-HA vector (a gift from Dr. Mark Christian’s lab) to generate pCS2-HA-SOX2. Then, the BamHI/SpeI fragment of HA-SOX2 was inserted into pSKII-Puro-2A-TRF2 plasmid [27] to replace TRF2. The HA-SOX2 sequence has been confirmed in-frame with the puro-2A sequence. Then, the PmeI/SpeI fragment of Puro-2A-HA-SOX2 was inserted into pLVTHM (Addgene #12247) [28] to replace the green fluorescent protein (GFP). Sox2 mutants were generated by site-directed mutagenesis of pCS2-HA-Sox2 using Q5 Site-Directed Mutagenesis Kit (New England Biolabs) following the manufacturer’s protocol with the appropriate primers (Additional file 1: Table S1). Lentivectors containing shSOX2 (Addgene, #26352, #26353) were obtained from Addgene, provided by Matthew Meyerson [29].

The Flag-OC4 expression plasmid was generated by inserting an amplified PCR fragment of OCT4 from OKSIM plasmid into the pCMV-Flag expression vector. OCT4 regulatory fragment was isolated from OCT4-EGFP plasmid [23] and inserted into pGL4.10 plasmid (Promega) to generate the 4-kb OCT4-driven luciferase reporter. The plasmid was then digested with EcoRI-EcoRV to remove both DE and PE for the generation of OCT4 mini promoter-driven reporter. OCT4-DE luciferase reporter was generated by inserting EcoRV/SphI fragment from the OCT4-EGFP into the OCT4 mini promoter-driven reporter construct, whilst the OCT4-PE reporter plasmid was constructed by inserting XhoI/NcoI fragment from the OCT4-EGFP into the PGL4.10.

PAX6-V5 expression vector was generated by replacing the Cidea cDNA from the pCDNA3.1-V5 backbone with the BamHI/XhoI PCR fragment containing PAX6 cDNA amplified from mRNA of hNPCs with specific primers (Additional file 1: Table S1). The BRN2-enh luciferase reporter was generated by inserting the KpnI/NheI fragment containing BRN2 enhancer amplified from genomic DNA of hESCs into the pGL4.23 vector (Promega).

### Transfection and lentiviral transduction

The transfections were performed with Lipofectamine LTX and PLUS reagent (Life Technologies) as described previously [9]. Briefly, 1–1.5 μg plasmid DNA and 1.6–2.5 μl lipofectamine were diluted in 50 μl of OptiMEM each and incubated for 5 min before being mixed and incubated for another 25 min. Meanwhile, cells were counted and 0.5–1 × 10^6^ cells were directly mixed with the DNA-lipofectamine mixture for 10 min. Together, they were transferred to a 12-well plate with growth media and grew overnight before medium change.

Lentiviral particles were produced in HEK293T cells using standard protocols [28]. Confluent hESCs were dissociated into single cells with accutase (Sigma) and one third of the cells were mixed with lentivirus in Matrigel-coated plate in MEF-CM with 10 μM ROCKi (Y-27632, R&D Systems). Cells were fed with fresh MEF-CM daily and selected with puromycin (2 μg/ml) 72 h post-infection.

### Quantitative reverse transcription PCR (qRT-PCR)

Total RNAs were extracted from cells using TRI reagent (Sigma). First-strand cDNA was synthesised with ProtoScript® II Reverse Transcriptase (NEB) and qRT-PCR was performed in a DNA Engine Opticon system (Bio-Rad) using SYBR Green Jumpstart Taq Ready Mix (Sigma) (Additional file 1: Table S1 for primers). For each experiment, RNAs were collected from at least two independent cell cultures and qRT-PCR were performed in triplets. Data were normalised to two housekeeping genes (β-ACTIN and RPL22) and presented as fold change to controls.

### Immunoblotting (IB), immunostaining, flow cytometry and cell sorting

Cells were lysed in cold radioimmunoprecipitation assay (RIPA) buffer (50 mM Tris-HCl, pH 8.0, 150 mM NaCl, 1% Nonidet-P40, 0.5% sodium deoxycholate and 0.5% SDS) containing protease inhibitor cocktail and 0.2 mM phenylmethanesulfonylfluoride (PMSF) (Sigma). Twenty micrograms proteins were resolved in SDS-polyacrylamide gel and transferred to polyvinylidene fluoride membranes and then probed with primary and secondary antibodies before chemiluminescent substrate reaction and exposure onto CL-XPosure film. Signals were quantified with ImageJ software, normalised to loading controls and presented as relative levels to controls. For immunostaining, cells were seeded onto Matrigel-coated Thermanox coverslips (Thermo Fisher Scientific) and fixed with 4% paraformaldehyde before being stained with indicated antibodies as described previously [9, 24]. The signal was visualised and captured with a Leica SP5 confocal microscope. Multiple images were captured and counted. Cells were stained as previously described [24] then analysed and sorted on Calibur, DIVA, or Aria flow cytometers (BD). All antibodies were with listed in Additional file 1: Table S2.

### Alkaline phosphatase-positive (AP+) colony formation assay

hESCs were dissociated into single cells in MEF-CM containing 10 μM Y-27632. Five hundred cells were seeded into a Matrigel-coated 12-well plate with medium change to MEF-CM 24 h later and daily thereafter for another 5 days. The cells were then fixed and stained using the Alkaline Phosphatase Detection Kit (Millipore-SCR004) following the manufacturer’s instruction.

### Co-immunoprecipitation (Co-IP)

Cells were lysed in buffer (40 mM HEPES pH 7.4, 5 mM EDTA, 0.05% 2-Mercaptoethanol, 10 mM NaCl, 0.5% NP40) supplemented with protease inhibitors and PMSF. One milligram of protein was incubated with an appropriate antibody for 90 min at 4 °C with rotation. Immunocomplexes were isolated using Protein-G conjugated Dynabeads® (Thermo Fisher Scientific) through a further 1-h incubation under the same conditions. Non-specific binding was removed prior to the elution by 2 × electrophoresis buffer. Elutes were then subjected to immunoblotting and the co-immunoprecipitation was examined using relevant antibodies.

### ChIP-Seq and single-cell RNA-Seq data acquisition and analysis

Published ChIP-Seq datasets used in the studies are GSE32465 and GSE69646 of OCT4 in hESCs [30, 31], GSE49404 and GSE69479 of SOX2 in hESCs and hNPCs [32, 33] and GSE24447 of H3K27ac in hESCs and hNPCs [34], and Pax6 ChIP-Seq dataset in hNPCs was provided by Professor Stanton’s lab in Singapore [25]. Sequencing reads were downloaded from the Gene Expression Omnibus (GEO) [35] as SRA files and converted to fastq format using the SRA tool kit (www.ncbi.nlm.nih.gov/books/NBK158900/). Read quality was assessed using FastQC (www.bioinformatics.babraham.ac.uk/projects/fastqc/). Read filtering and trimming was performed with Trimmomatic [36]. Reads were aligned to the hg19 human reference genome using Bowtie2 [37]. Statistically significantly enriched peaks were identified using MACS2 [38] by comparing the read densities of the experiment to the relevant control. If the shift size was estimated at < 70, peak calling was repeated without modelling, and the shift size was set based on the fragment size selection stated in the publication associated with the ChIP-Seq experiment. For histone modification ChIP-Seq experiments, MACS2 was run with the “--broad” parameter.

Gene annotation and motif enrichment were performed using HOMER [39]. Gene ontology was performed with the PANTHER classification system [40] using the PANTHER GO-Complete Biological Process annotation dataset. *p* values were Bonferroni corrected for multiple hypothesis testing.

In the single-cell RNA-Seq analysis, processed and summarised read counts were downloaded from GEO (GSE86894). Cells that exhibited one or more reads for both the Oct4 and Pax6 genes were consider co-expressing.

### Luciferase reporter assay

Cells in 12-well plates were transfected with firefly and pRL-T7-renilla (Promega) plasmids at a ratio of 100:1 using Lipofectamine LTX as described above. The cells were harvested 48 h later and dispensed equally into a 96-well luminometer plate. Luciferase assay was performed using the Dual-Glo luciferase assay kit (Promega) following the manufacturer’s instruction, and the luminescence was measured in a Victor II luminometer (Perkin Elmer). Firefly luminescence readings were normalised with the corresponding renilla readings. Data presented are mean ± SD from three independent transfection experiments.

### Statistical analysis

For qPCR and luciferase assay, unpaired, two-tailed Student *t* test was used with at least three independent biological samples to determine the statistical significance. For the processing of ChIP-Seq datasets, please refer to the “ChIP-Seq and single-cell RNA-Seq data acquisition and analysis” section.

## Results

### Deficiency of SOX2 results in the loss of both hESC and neural progenitor properties

**Fig. 1:**
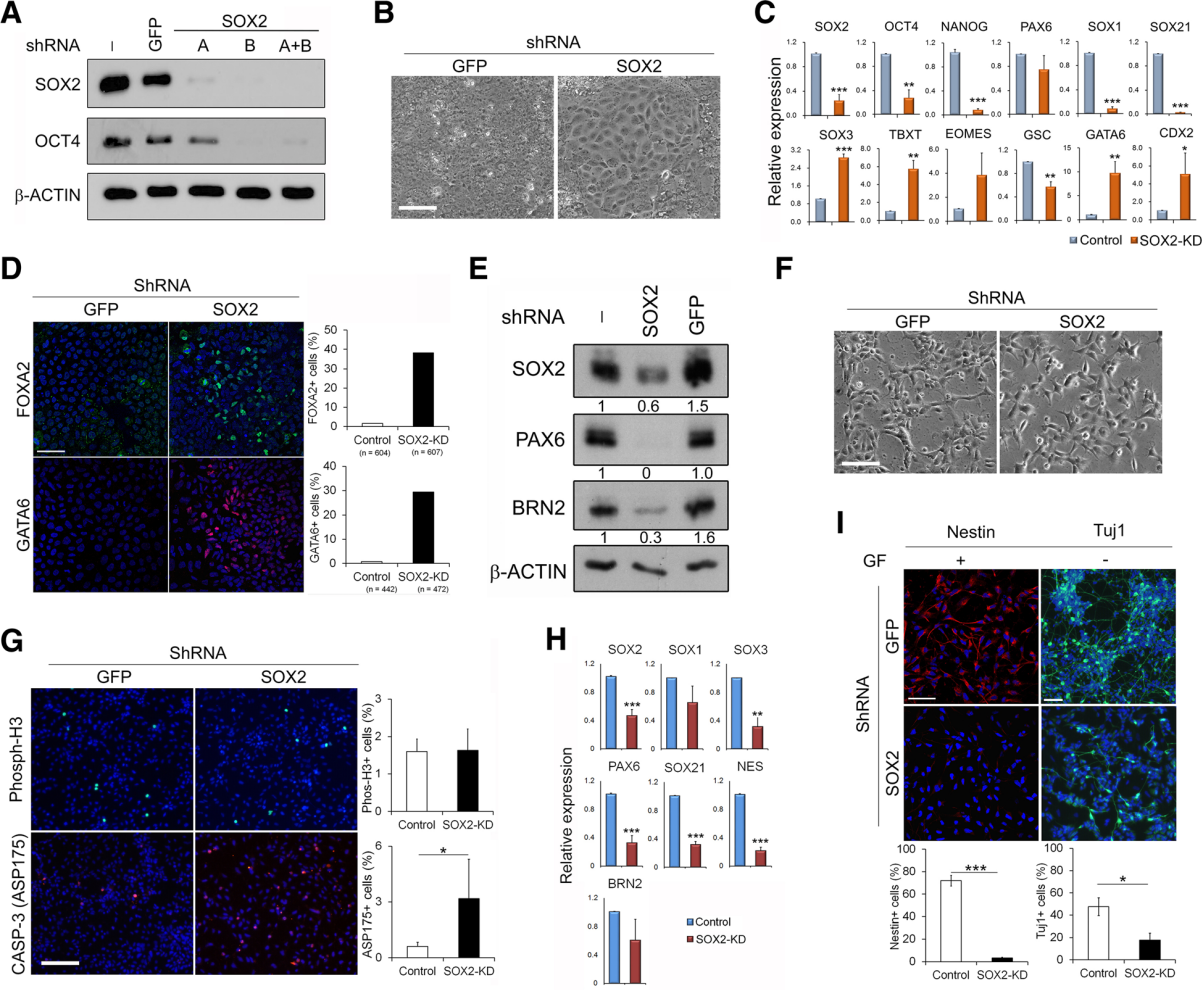
SOX2 knockdown in hESCs and NPCs affect their properties. H1 hESCs (**a**–**d**) and their derived neural progenitors (**e**–**i**) were infected by either SOX2-shRNA or control-shRNA and analysed 7 days later unless indicated. **a** Representative immunoblot showing knockdown of SOX2 with indicated SOX2 shRNA in hESCs. **b** Representative images comparing morphology in control and SOX2-knockdown (KD) hESCs with shRNA(A+B). Scale bar = 100 μm. **c** Transcript levels by qRT-PCR in control and SOX2-KD hESCs. Data are presented as mean ± SD (from 3 independent shRNA transduction experiments of triplicate PCRs for each sample). **d** Immunostaining with FOXA2 and GATA6 antibodies in control and SOX2-KD hESCs. Left—representative images, scale bar = 50 μm; right—the percentage of positive cells from the numbers of cells counted shown below. **e** Immunoblot showing reduced PAX6 and BRN2 expression in SOX2-KD NPCs with quantification shown under. **f** Representative phase-contrast images of control and SOX2-KD NPCs. Scale bar = 100 μm. **g** Immunostaining with indicated antibodies in control and SOX2-KD NPCs. Left—representative images, scale bar = 50 μm; right—quantitative analysis from 3 independent experiments as in **c** with > 3000 cells counted in each sample. **h** mRNA expression by qRT-PCR in control and SOX2-KD NPCs. Data are shown as in **c**. **i** Immunostaining with nestin and Tuj1 antibodies. Tuj1 staining was performed after withdrawal of growth factors from NPCs for 7 days. Upper—representative images, scale bar = 50 μm; lower—quantitative analysis from 3 independent experiments with > 2000 cells counted in each sample. *p < 0.05; **p < 0.005 and ***p < 0.0005 by Student *t* test

To validate the function of SOX2 in hESCs, we firstly interrupted *SOX2* expression in self-renewing hESCs by lentiviral-transduced shRNAs in H1 and H7 hESCs [29]. Upon knockdown of SOX2 by various shRNAs (Fig. 1a), hESCs exhibited dramatic changes in their morphology within 7 days from tight hESC colonies to flat, endoderm-like cells (Fig. 1b). Gene expression analysis revealed significant downregulation of pluripotent genes, such as *OCT4* and *NANOG*, and concomitant upregulation of mesoderm and endoderm markers (Brachyury (*TBXT*), *EOMES*, *GSC*, *GATA6* and *FOXA2*) in the SOX2-knockdown (SOX2-KD) cells (Fig. 1c, d; Additional file 1: Figure S2A). In contrast, neural genes, including *PAX6*, *SOX1* and *SOX21*, displayed a reduction in expression with the notable exception of *SOX3*. The alterations in gene expression profile were consistent with the observed morphological changes and demonstrated that hESCs were mainly differentiated into an endoderm-like cell type in the absence of SOX2. These results reiterate that SOX2 is an essential factor for the maintenance of hESC cultures similar to that in mESCs.

As SOX2-KD resulted in the loss of pluripotency in hESCs, this precluded us to address the effect of SOX2 on hESC neural specification using these depleted cells. Thus, aiming to explore the importance of SOX2 in neural differentiation, we next examined the effect of SOX2-KD in hESC-derived proliferating NPCs, 4–6 weeks after hESC neural differentiation (Fig. 1e). SOX2-KD in NPCs did not dramatically alter their cell morphology and proliferation index as assayed by phospho-H3 staining but significantly raised the incidence of apoptosis, as evidenced by an increase of Caspase-3 activation (Fig. 1f, g). More importantly, SOX2-KD in NPCs resulted in an overall reduction in the expression of neural progenitor markers (Fig. 1e, h, i) and a significant decline (from 48 to 18%) in Tuj1-positive neurons upon further differentiation into post-mitotic neurons (Fig. 1i), demonstrating an important role of SOX2 in sustaining the survival and neuronal differentiation potential of human NPCs. Taken together, our findings suggest that SOX2 is a vital factor for both the maintenance of hESC pluripotency and NPC neural identity.

### Overexpression of SOX2 in hESCs resulted in differential effects depending on culture conditions

**Fig. 2:**
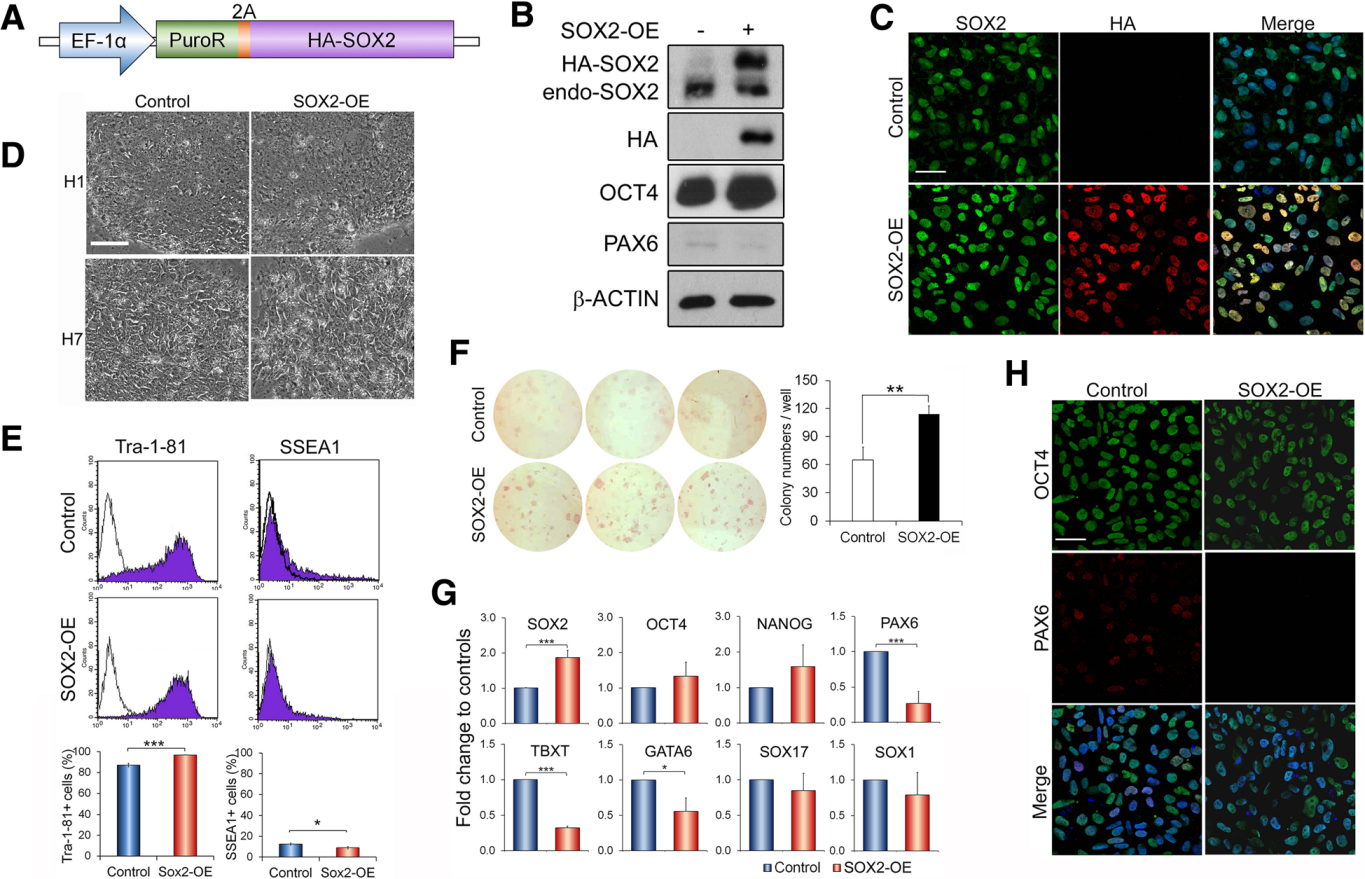
Overexpression of SOX2 inhibits spontaneous differentiation in hESC culture under self-renewal conditions. All analyses were done around 30 days post-transduction in H1 hESCs unless indicated. **a** Schematic of SOX2 overexpression construct and vector containing PuroR only as the control. **b** Immunoblot of cell lysate isolated from control and SOX2-overexpressing (SOX2-OE) hESCs with indicated antibodies. Both HA-SOX2 and endogenous Sox2 are indicated. **c** Representative images of indicated hESCs immunostained with SOX2 and HA antibodies. Scale bar = 50 μm. **d** Phase-contrast images of indicated hESCs in self-renewal cultures. Scale bar = 100 μm. **e** Flow cytometry analysis of Tra-1-81 and SSEA1 in the indicated hESCs. The upper panels are representative flow cytometry images and the lower histograms are the quantification as mean ± SD from three independent transductions. ***p < 0.001 and *p < 0.05 by Student *t* test. Similar results were also obtained many passages later. **f** Colony formation assay with alkaline phosphatase staining in SOX2-OE and control hESCs (> 2 months post-transduction). Representative images of three different OE and their corresponding control cell lines (left) and their counting of triplicate for each line (right). **p < 0.01. **g** qRT-PCR analysis of gene expression in the indicated hESCs. Data are normalised to control hESCs and presented as mean ± SD from three independent OE experiments of triplicate PCRs for each. **h** Immunostaining of indicated hESCs with OCT4 and PAX6 antibodies. Scale bar = 50 μm

To further explore the role of SOX2 in hESCs, HA-tagged SOX2 (Fig. 2a) was stably expressed into both H1 and H7 hESCs (SOX2-OE), and the resulting cells exhibited up to over twofold increases in total SOX2 protein without significant changes in endogenous SOX2 expression (Fig. 2b, c). These SOX2-OE cultures presented typical hESC colonies with no noticeable sign of differentiation under our routine self-renewal conditions (Fig. 2d). Thus, a detailed analysis was focused on H1 cells. Close inspection revealed that SOX2-OE cultures actually encompassed a lower number of spontaneously differentiated cells than control hESCs, with an average of 96% versus 87% Tra-1-81 (undifferentiated)-positive cells and conversely 9% versus 13% of cells staining positive for SSEA1 (differentiated) (Fig. 2e). As further evidence, higher numbers of newly formed undifferentiated hESC colonies were observed in SOX2-OE relative to control cells as assayed by alkaline phosphatase-positive colony formation (Fig. 2f). Consistently, the pluripotent genes *OCT4* and *NANOG* exhibited slightly increased transcript levels in SOX2-OE relative to control hESCs, whereas many lineage-specific genes including *PAX6*, *TBXT*, *GATA6*, *SOX17* and *SOX1* showed reduced transcript expression in these cells (Fig. 2g), as confirmed at the protein level for OCT4 and PAX6 (Fig. 2b) with heterogeneity (Fig. 2h, Additional file 1: Figure S2B). The slight increase of *OCT4* expression in SOX2-OE hESCs was not merely resulted from the decrease of spontaneous differentiation as it could also be recapitulated in sorted Tra-1-81-positive, undifferentiated hESCs (Additional file 1: Figure S2C). These data indicate that a high level of SOX2 inhibits spontaneous differentiation and enhances self-renewal of hESCs under self-renewal culture conditions.

**Fig. 3:**
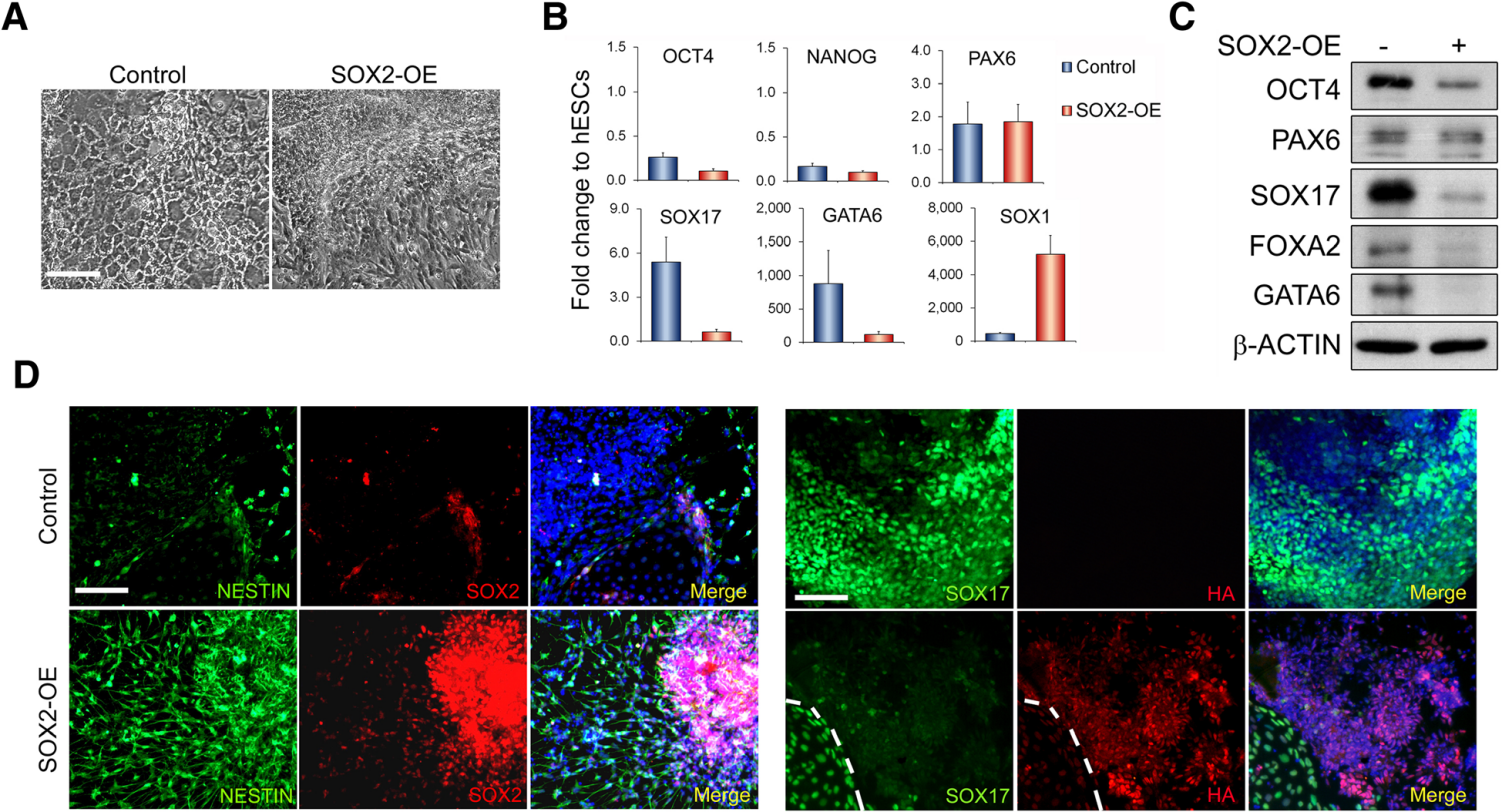
Overexpression of SOX2 predisposes hESCs to neural differentiation in N2B27 media. **a** Representative images of control and SOX2-OE H1 hESCs cultured for 14 days in the N2B27 medium. Scale bar = 100 μm. **b** mRNA expression in control and SOX2-OE cells of **a** by qRT-PCR as relative to undifferentiated control hESCs. Data are presented as mean ± SD from six PCR tests of two independent biological samples. **c** Immunoblot of cell lysates isolated from **b** with indicated antibodies. **d** Immunostaining of cells as in **b** showing more nestin and less SOX17-positive cells in SOX2-OE cultures than controls. The area containing positive SOX17 signals in SOX2-OE culture is marked with dotted lines to show the absence of transgenic SOX2

Since overexpressing SOX2 revealed an enhancement on self-renewal of hESCs, we asked whether this high level of SOX2 is also capable of supporting hESC self-renewal in either unconditioned KSR medium or chemically defined N2B27 medium, which are either the base medium for our hESC cultures or the base medium for hESC neural differentiation [7, 23]. Within 3 weeks, both SOX2-OE and control hESCs exhibited morphological changes in which control cells showed more flattened, epithelial endoderm-like phenotype, whilst SOX2-OE cells displayed highly packed cells with neural progenitor features, particularly obvious in the N2B27 cultures relative to KSR medium: Fig. 3a, Additional file 1: Figure S3A). In line with their morphological appearances, both control and transgenic cells revealed reduced OCT4 and NANOG mRNAs, relative to the control hESCs under self-renewal conditions (Fig. 3b, Additional file 1: Figure S3B). However, SOX2-OE and control cells exhibited distinct patterns in lineage-specific gene expression. SOX2-OE cells expressed considerably lower levels of endoderm marker genes (*TBXT*, *SOX17*, *GATA6* and *FOXA2*) and higher level of the neural gene *SOX1* than controls (Fig. 3b, Additional file 1: Figure S3B, C), suggesting a preference for neural lineage specification of SOX2-OE cells upon exiting from pluripotency. Moreover, SOX2-OE hESCs grown under N2B27 conditions also revealed more neural NESTIN and less endodermal SOX17 signals than control cultures in immunostaining (Fig. 3d). Although positive SOX17 signals were sporadically detected in SOX2-OE cultures, they were restricted to a limited number of cells that have also lost SOX2 transgene expression (Fig. 3d, marked region), possibly due to epigenetic-induced transgene silencing. Interestingly, whilst PAX6 seemingly exhibited comparable expression levels in both SOX2-OE and control cultures under N2B27 conditions (Fig. 3b, c), the ratio of PAX6 over OCT4 was considerably higher in SOX2-OE cells than in controls, which could impact on lineage specification.

Altogether, these results demonstrated that SOX2 has a dual functional role in hESCs with high levels of SOX2 potentiating hESC self-renewal under pluripotency culture conditions whilst promoting neural differentiation, possibly at the expense of mesendoderm cell fates, at least as tested in KSR and N2B27 culture conditions.

### PAX6 is a SOX2-interacting partner in the early neural differentiation

**Fig. 4:**
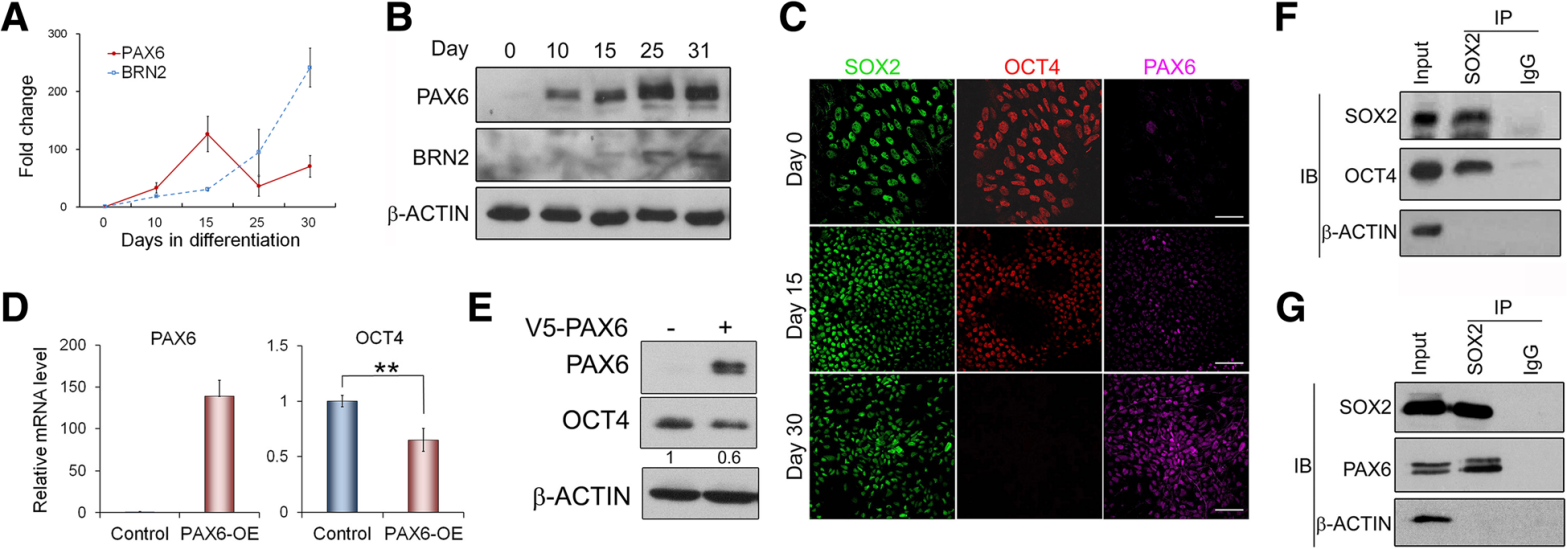
SOX2 interacts with OCT4 and PAX6 in hESCs and hNECs. **a** Dynamic mRNA expression of PAX6 and BRN2 by qRT-PCR during the neural differentiation of H1 hESCs as shown in Additional file 1: Figure S1. **b** Immunoblot of PAX6 and BRN2 protein expression in cells of **a**. **c** Immunostaining with indicated antibodies during the neural differentiation of hESCs. Days of differentiation are indicated. Scale bar = 50 μm. **d** qRT-PCR analysis of H1 hESCs 48 h after ectopic PAX6 expression. Data are presented as mean ± SD from three transduction experiments. **p < 0.01, ***p < 0.0005. **e** Immunoblot showing the effect of PAX6 overexpression on OCT4 in hESCs with quantification shown below. **f** Co-IP showing that SOX2 interacts with OCT4 in undifferentiated hESCs. **g** Co-IP showing that SOX2 interacts with PAX6 in hESC-derived neural epithelial cells

We propose that these diverse roles of SOX2 in hESCs influenced by culture conditions are likely attributed to the expression of SOX2-interacting partners, which shaped the formation of different SOX2-interacting complexes and impacted on SOX2 transcriptional regulatory functions [19]. SOX2 interacts with OCT4 in undifferentiated hESCs to activate pluripotent gene expression programme. Upon changing to non-conditioned KSR or N2B27 culture conditions, OCT4 expression might be reduced possibly with the increase of other SOX2 binding partners, including neural transcription factors (NTFs). This might affect SOX2-OCT4 interaction and their target genes. To identify SOX2-interacting NTFs, we surveyed published reports and noticed two likely NTF candidates: Pax6 and Brn2. Both factors are highly expressed in the central nervous system (CNS) and capable of binding to Sox2 [5, 22, 41, 42]. Detailed analysis of their expression revealed that both factors were hardly detected in undifferentiated H1 hESCs. However, upon neural differentiation, PAX6 was considerably induced at the early initiation stage, whilst BRN2 was only substantially upregulated after hESCs had differentiated to NPCs when OCT4 expression was significantly downregulated (Fig. 4a, b; Additional file 1: Figure S1C,D). Surprisingly, PAX6 protein levels continued to increase after day 25 of neural differentiation despite its mRNA levels being already downregulated, possibly pointing to translational or post-translational regulation as the experiments have been repeated several times with similar outcomes. These findings indicate that PAX6 is upregulated prior to BRN2 during hESC neural differentiation and hence might have a more crucial role in the initiation of neural differentiation.

Upon hESC neural differentiation, OCT4 and PAX6 expression quickly became inversely correlated (Fig. 4c), yet sporadic neural differentiating cells were observed to co-express the two factors at an early stage of neural differentiation by immunostaining [24] and single-cell RNA-Seq analysis (Additional file 1: Figure S4A) [43]. We thus reckoned that upregulation of PAX6 as a result of inhibiting dual-Smads pathways could repress OCT4 expression in the early human neural differentiating cells, leading to neural progression. However, given the dynamic nature of these events, only a small number of cells co-expressing OCT4 and PAX6 might be captured. Accordingly, we found that transiently expressing PAX6 in hESCs was sufficient to significantly reduce both OCT4 mRNA and protein levels within 48 h post-transduction (Fig. 4d, e), which is consistent with the previous report [22]. Furthermore, SOX2 co-immunoprecipitation (Co-IP) in undifferentiated hESCs and hESC-derived neural epithelial cells (NECs) showed that SOX2 was capable to interact with PAX6 in NECs and with OCT4 in hESCs (Fig. 4f, g). Consistent with the report that mouse Pax6 interacts with Sox2 using the same Sox2 interface as the Sox2-Oct4 binding [44], mutations in human SOX2-HMG domain were shown to similarly affect SOX2 interaction with OCT4 and PAX6 (Additional file 1: Figure S4B-D). Moreover, the expression of the increasing amount of PAX6 revealed a reduction in SOX2-OCT4 interaction and an increase in SOX2-PAX6 interaction (Additional file 1: Figure S4E). This suggests that PAX6 induction might also affect SOX2-OCT4 interaction and transcriptional regulatory function.

Altogether, our results demonstrate that PAX6 is rapidly upregulated upon neural differentiation in hESCs and is able to interact with SOX2. This newly formed SOX2-PAX6 complex may function to inhibit OCT4 expression and affect the transcriptional states of cells, leading to hESC neural differentiation.

### SOX2-OCT4 and SOX2-PAX6 regulate different target genes

**Fig. 5:**
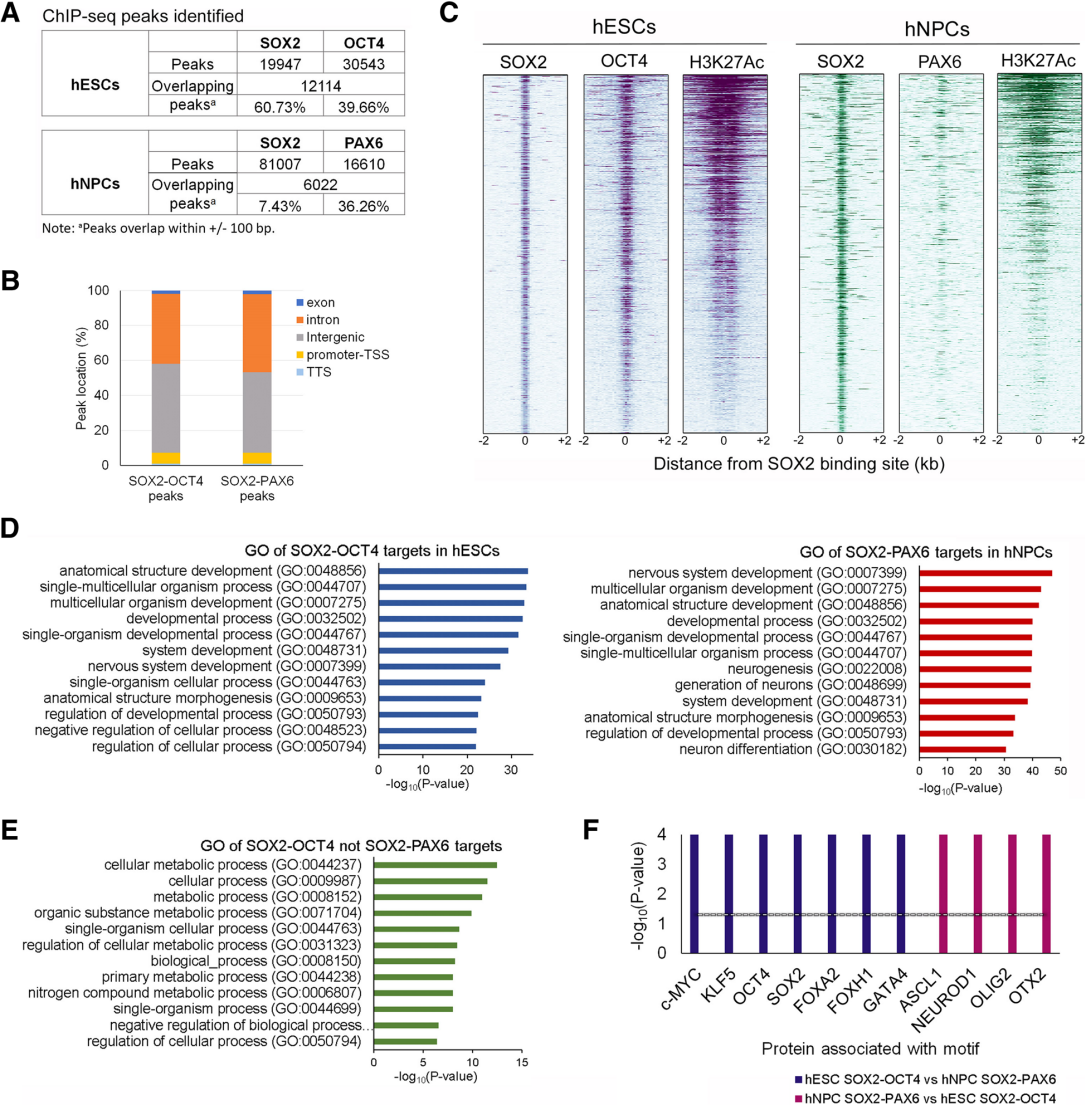
Analysis of SOX2-OCT4 and SOX2-PAX6 targeting genes from ChIP-Seq datasets. **a** Identification of SOX2, OCT4 and PAX6 peaks from ChIP-Seq datasets in hESCs and hNPCs. **b** The genomic distribution of SOX2-OCT4 and SOX2-PAX6 overlapping peaks in hESCs and hNPCs, respectively. TSS, transcription start site; TTS, transcriptional terminal site. **c** Heatmap of SOX2 peaks in hESCs (left panel) and hNPCs (right panel) from ChIP-Seq datasets centred on the SOX2-binding sites (± 2 kb) and ordered top to bottom by signal intensity. OCT4, PAX6 and H3K27ac ChIP-Seq signals associated with the corresponding Sox2-binding sites in the two cell types are shown. **d** Top 10 biological functions in GO analysis of the nearest genes of SOX2-OCT4-H3K27ac and SOX2-PAX6-H3K27ac overlapping regions in hESCs and hNPCs, respectively. **e** Top 10 biological functions in GO analysis of the nearest genes of SOX2-OCT4-H3K27ac overlapping regions in hESCs, which are not bound by SOX2-PAX6 in hNPCs. **f** SOX2-OCT4 (blue bars)- and SOX2-PAX6 (maroon bars)-enriched motifs are associated with transcription factors of different lineages. The black line indicates the background level

To further investigate the function of SOX2-OCT4 and SOX2-PAX6 complexes in hESCs and during their neural differentiation, we next thought to explore their transcriptional regulatory functions by analysing publically available chromatin immunoprecipitation-sequencing (ChIP-Seq) datasets of OCT4 in hESCs [30, 31], SOX2 in hESCs and derived NPCs [32, 33] and PAX6 in hESC-derived NECs [25] alongside the histone mark H3K27ac [34]. Although these datasets are from different laboratories and experiments, they were generated from similar hESC culture conditions and the same neural differentiation protocols, and they are the most closely associated data available. All the datasets were analysed for motif enrichment prior to further analysis, and the top results from all OCT4 and SOX2 ChIP-Seq data were all OCT4 and SOX2 motifs, which certify the specificity of these datasets. PAX6 dataset did not show specific motif enrichment as previously reported [25]. SOX2 was detected to bind to 19,947 and 81,007 sites genome wide in hESCs and hNPCs, respectively, with 61% of these sites overlapping with OCT4 in hESCs and 7.4% with PAX6 in hNPCs (Fig. 5a, Additional file 1: Figure S5A,B). As expected, both SOX2-OCT4 and SOX2-PAX6 were predominantly detected at intergenic and intronic regions (> 90%) rather than promoter transcription start sites (TSS) (Fig. 5b). Heatmap analyses further revealed an evident association in the localization of SOX2 and OCT4 binding at these sites in hESCs, which was correlated with the active enhancer marker H3K27ac. This confirmed that SOX2 and OCT4 coordinately regulate the expression of their target genes. A similar pattern was also observed for SOX2 and PAX6 in hNPCs, although SOX2 might independently cooperate with other neural TFs in these committed neural progenitors (Fig. 5c). Assuming that SOX2-OCT4 and SOX2-PAX6 are more likely to regulate the nearest genes to their binding sites in hESCs and hNPCs, respectively, we carried out Gene Ontology analysis on identified affiliated gene targets. This analysis revealed that although both sets of genes were commonly associated with developmental processes, SOX2-PAX6 targets were more specifically involved in the development of the nervous system (Fig. 5d). Interestingly, 67% (2246/3347 genes) of the SOX2-PAX6-H3K27ac targets in hNPCs were also occupied by SOX2-OCT4 in hESCs despite not all of the sites being associated with H3K27ac in this cell type. In contrast, only 38% (1988/6261 genes) of SOX2-OCT4-H3K27ac sites in hESCs were targeted by SOX2-PAX6 in hNPCs (Additional file 1: Figure S5C). This denotes that many SOX2-PAX6 targets in hNPCs might be pre-marked by SOX2-OCT4 in hESCs regardless of their activation status, whereas the majority of the SOX2-OCT4 active targets in hESCs are not being regulated by SOX2-PAX6 in hNPCs. Inspection of the latter group, interestingly, revealed a significant enrichment of genes involved in metabolic processes (Fig. 5e), suggesting that SOX2-OCT4 might have a crucial function in the maintenance of pluripotency via regulating cell metabolism. Differential regulatory functions for SOX2-OCT4 and SOX2-PAX6 were also supported by motif enrichment analysis, by which SOX2-OCT4 bound regulatory regions were confirmed to encompass several DNA binding motifs of other pluripotency-associated factors, such as c-MYC and KLF5, as well as key mesendoderm-associated transcription factors, including FOXA2, FOXH1 and GATA4. In contrast, SOX2-PAX6 regulatory regions were the most closely associated with NTF motifs, as exemplified by OTX2, ACL1, OLIG2 and NEUROD (Fig. 5f). To further validate the functional implication of SOX2-PAX6 binding, we analysed the mRNA expression of their target genes in early neural differentiating hESCs using our previous RNA-Seq data [45]. A total of 1631 SOX2-PAX6 target transcripts were detected in our dataset with ~ 30% showing upregulation and ~ 20% showing downregulation. GO analysis of both gene sets revealed that the upregulated genes are more specifically involved in neural differentiation, whereas the downregulated ones are associated with generic terms of organismal development (Additional file 1: Figure S5D). These data further support that the SOX2-PAX6 complex has a critical function in activating neural gene expression, hence implying an important role in neural differentiation of hESCs.

### SOX2-PAX6 complex differentially regulates OCT4 and BRN2 expression

**Fig. 6:**
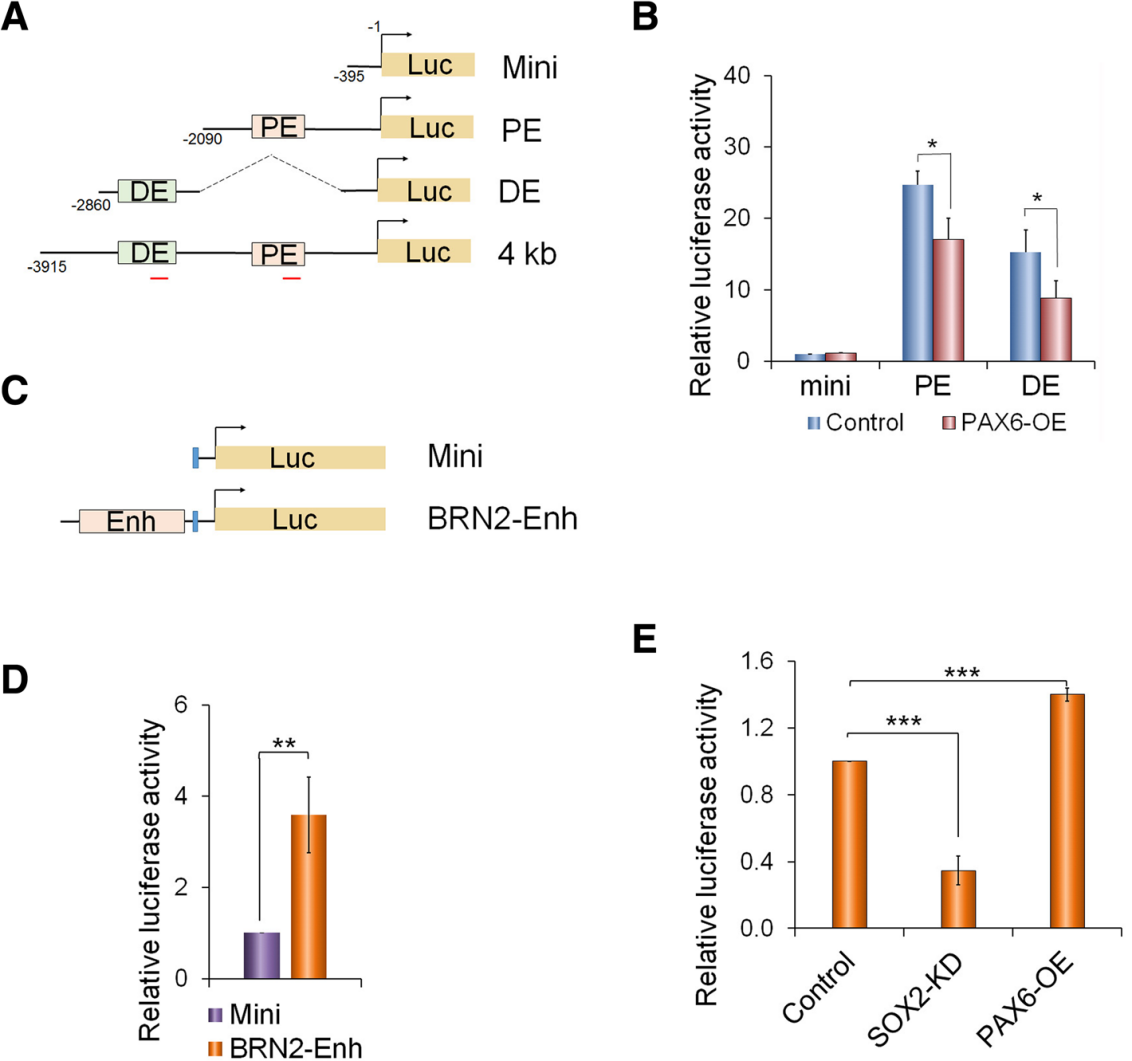
PAX6-SOX2 differentially regulate OCT4 and BRN2 expression. **a** Schematic of OCT4-luciferase reporter constructs. PE and DE, proximal and distal enhancer regions, respectively; red bars—qPCR amplifying fragments in the ChIP-PCR. **b** Significant reduction of OCT4-luciferase activities in H1 hESCs co-transfected with PAX6 expressing vector. Histograms: quantitative analysis (*n* = 3) and *p < 0.05; representative immunoblot of PAX6 overexpression in the inserted images. **c** Schematic of BRN2-enhancer (BRN2-enh) luciferase reporter constructs. **d** BRN2-luciferase reporter assay in hNPCs. **e** Effect of SOX2-knockdown (SOX2-KD) and PAX6 overexpression (PAX-OE) on BRN2-enh luciferase activities in hNPCs. ***p < 0.0005 (*n* = 3)

Given that ectopically expressing PAX6 into undifferentiated hESCs quickly and significantly reduced OCT4 expression (Fig. 4d, e), it is plausible that PAX6 or the SOX2-PAX6 complex may directly bind to OCT4 enhancer to repress OCT4 expression. To directly address this, a luciferase reporter assay was carried out in hESCs, in which luciferase expression was driven by various OCT4 regulatory elements (Fig. 6a). As expected, the OCT4 enhancer-driving reporters exhibited significantly higher luciferase activities compared to the mini-promoter among which the OCT4 proximal enhancer (PE)-luciferase produced the highest activities (Additional file 1: Figure S6A), confirming that OCT4 is mainly regulated by its PE element in hESCs [46, 47]. When ectopically expressed in hESCs, PAX6 was shown to significantly reduce the OCT4 enhancer-driven luciferase activities (Fig. 6a). However, it is unclear whether this repression is by direct binding of PAX6 on OCT4 enhancer or an indirect suppression via other factors. Although the PAX6 ChIP-Seq data in hNPCs did not detect enrichment on OCT4, it could not be completely excluded the possibility that PAX6 might bind to OCT4 enhancers to repress them by inducing epigenetic remodelling, e.g. DNA methylation, which then makes the enhancers inaccessible for further binding. To support this view, SOX2 ChIP-Seq in hNPCs also did not detect the binding on OCT4 enhancers either [33]. Since this dynamic process occurs rapidly upon PAX6 is upregulated, it is very difficult for us to test this experimentally.

Interestingly, we noticed from our analysis of the ChIP-Seq datasets that SOX2 and PAX6 are co-enriched in human NPCs at a region located ~ 200 kb upstream of its nearest gene, *POU3F2*, encoding BRN2 (Additional file 1: Figure S6B). This region was recently identified by Promoter Capture Hi-C analysis as an enhancer region of *POU3F2*, which exhibited regulation on reporter gene expression in the CNS of mouse embryos [48]. To verify the regulatory function of SOX2 and PAX6 in this region, a BRN2-luciferase reporter assay, in which this enhancer sequence was inserted upstream of the *tk* mini-promoter, was performed in the hESC-derived NPCs (Figs. 6c, d). Upon knockdown of SOX2 in the NPCs, the BRN2-luciferase activity was dramatically reduced, whilst it was significantly increased after overexpressing PAX6 (Fig. 6e). As SOX2 interacts with PAX6 in hNECs (Fig. 4e) and their binding peaks overlap at this region, it is likely that both SOX2 and PAX6 jointly activate BRN2 expression. This is also supported by the observations that BRN2 expression lagged behind PAX6 upregulation during hESC early neural differentiation (Fig. 4a, b). Our analysis on SOX2-PAX6 cooperative binding motifs based on published datasets did not identify with confidence a consensus PAX6 motif, which is in line with other reports [25, 49]. However, this impeded us for motif mutation analysis. Nonetheless, taken together, our data suggest that SOX2 and PAX6 together play a critical role in the regulation of OCT4 and BRN2 expression, inhibiting OCT4 expression and activating BRN2 expression, therefore promoting hESC neural differentiation.

## Discussion

The notion that the core pluripotency factors Oct4 and Sox2 play a key role not only in maintaining cell pluripotency but also in the determination of lineage specification [12] has attracted great attention from stem cell researchers as this idea bridges the mechanistic transition from a pluripotent stem cell (PSC) to a lineage-specific cell type. Using genetic manipulations, here, we provide direct evidence that SOX2 has a dual function in hESCs: maintaining the pluripotency and promoting their neural differentiation. In the absence of SOX2, hESCs and hESC-derived hNPCs are unable to retain their pluripotent and neural progenitor properties, respectively. Although we also observed a significant upregulation of SOX3 mRNA in SOX2-knockdown hESCs as previously reported [4], this increase of SOX3 did not compensate for the SOX2 deficiency to maintain hESCs in a pluripotent state in our experiments, an observation that differs from a previous report [4], but is in agreement with the others [17, 18]. This discrepancy could be attributed to several factors, such as culture conditions, degree of SOX2 knockdown and even pluripotency status. Our results indicate that SOX2 is an essential factor in hESCs, as it is in mESCs, for the maintenance of their self-renewal and pluripotency.

Interestingly, our experiments showed that overexpression of SOX2 in hESCs leads to two distinct phenotypes, depending on their culture environments. Under self-renewing conditions, SOX2 overexpression increases OCT4 expression, represses spontaneous differentiation and enhances hESC self-renewal; whereas upon release from self-renewing conditions, high levels of SOX2 result in activation of neural genes, repressing mesendoderm markers and promoting hESC neural differentiation. These results are in agreement with the previous reports in mESCs [16] and in hESCs [4] and further confirm that SOX2 has a dual functional role in PSCs.

Given that culture environments greatly affect the function(s) of SOX2 in hESCs, and that SOX2 requires interacting partners for its transcriptional activity, we anticipated that the outcome of SOX2 action might be determined by its key interacting partners [19, 50]. In addition to OCT4, the well-studied SOX2 partner in pluripotent hESCs, we identified PAX6 as an important SOX2 partner for hESC neural initiation based on the following evidence: (1) activation of PAX6 is an early event during hESC neural differentiation (Fig. 4), and it is also expressed in the single-layered NECs in early human embryos [22]; (2) PAX6 and SOX2 are expressed in the same NEC region during early human embryonic brain development, and PAX6 is able to interact with SOX2 in a similar manner as OCT4 (Additional file 1: Figure S4) [44]; (3) in the absence of PAX6, hESC neural differentiation is considerably impeded, whereas ectopic expression of PAX6 in hESCs results in neural ectoderm formation even under hESC self-renewing culture conditions [22]; (4) expression of PAX6 in hESCs significantly reduces OCT4 expression (Figs. 4 and 6); and (5) SOX2-PAX6 target genes are mainly associated with neural differentiation and nervous system development (Fig. 5). Therefore, we deem that SOX2-PAX6 complex might have a consolidating role during neural differentiation by promoting pre-neuroectoderm cells generated via inhibition of dual-Smads pathways [51, 52] to become neural progenitors. The role of the complex would include enhancing neural gene expression and inhibiting genes associated with pluripotency and mesendoderm lineages, although the mechanisms that induce locus-binding specificity of SOX2-PAX6 remains elusive due to the lack of specific binding motif of PAX6 [25]. Given that PAX6 ectopic expression was shown to enforce hESC neural differentiation [22], SOX2-PAX6 might also play a role in driving hESC neural differentiation if sufficient PAX6 is expressed.

Noteworthy, in genome-wide ChIP-Seq analysis, over 60% of the SOX2-PAX6-bound regions in hNPCs are also bound by SOX2-OCT4 in hESCs (Additional file 1: Figure S5C), which is in line with the previous finding [53], indicating that these regions may be pre-marked in hESCs prior to their timely activation upon differentiation. In addition, we noticed that only a small proportion of SOX2-binding regions in hNPCs are co-occupied by PAX6, whilst a considerable number of SOX2-binding sites are co-bound by OCT4 in hESCs. This might be due to the fact that PAX6 is mainly required to interact with SOX2 transiently for neural initiation by suppressing pluripotent genes (e.g. OCT4) and activating other NTFs (e.g. BRN2). Upon committing to neural differentiation, newly activated NTFs can, in turn, interact with SOX2 to specify different types of NPCs. Alternatively, unidentified NTFs might also function similarly to PAX6 as SOX2 partners for neural initiation eliciting the formation of distinct SOX2 complexes and hence the concomitant generation of different NPC subpopulations. Indeed, 14 different types of neural progenitors are identified from hESC neural differentiation using single-cell RNA-Seq [43].

## Conclusion

Taken together, our study demonstrates that Sox2 is a key factor in controlling PSC cell fate determination in humans as that in mice. The interaction of Sox2 with different partners dictates its function as either a pluripotent factor or a neural differentiation promoter. OCT4 is the key SOX2 partner for hESC pluripotency, whilst PAX6 is an important SOX2 partner for determining hESC neural specification after the exit of pluripotency. This study provides important experimental evidence to support the dual function of SOX2 as a pluripotent factor as well as a lineage specifier and to unveil a possible underlying mechanism for this SOX2 function.

## Additional file

Additional file 1: Figure S1. Distinct dynamic expression of SOX2 and OCT4 during hESC differentiation. Related to the introduction and Fig. 4. Figure S2. Effects of SOX2 expression in hESCs. Related to Fig. 1 and Fig. 2. Figure S3. SOX2-OE hESCs cultured in KSR medium. Related to Fig. 3. Figure S4. OCT4 and PAX6 co-express transiently in early neural differentiation and bind to the same SOX2 HMG domain motif. Related to Fig. 4. Figure S5. Analysis of SOX2-OCT4 and SOX2-PAX6 ChIP-Seq datasets. Related to Fig. 5. Figure S6. OCT4 and BRN2 enhancers. Related to Fig. 6. Table S1. Primers used in the study. Related to Figs. 1, 2, 3 and 6 and Figures S1, S3-S4. Table S2. Antibodies used in the study. Related to Figs. 1, 2, 3, 4 and 6 and Figure S1-S2 and S4. (PDF 1426 kb)

## Abbreviations

ChIP-Seq: Chromatin immunoprecipitation sequencing
CNS: Central nervous system
Co-IP: Co-immunoprecipitation
ESCs: Embryonic stem cells
hESCs: Human ESCs
KD: Knockdown
KSR: Knockout serum replacement
MEF-CM: mouse embryonic fibroblast - conditioned medium
mESCs: mouse ESCs
NECs: Neural epithelial cells
NPCs: Neural progenitor cells
OE: Overexpression
PE: Proximal enhancer
PSCs: Pluripotent stem cells
TFs: Transcription factors
TSS: Transcription start site
