# Supplementary material for: OCT4 and PAX6 determine the dual function of SOX2 in human ESCs as a key pluripotent or neural factor
Source: Stem Cell Res Ther. 2019 Apr 18;10:122. doi: 10.1186/s13287-019-1228-7 (PMC6471829; doi:10.1186/s13287-019-1228-7)
Supplement: Supplementary file 1 — Figure S1. Distinct dynamic expression of SOX2 and OCT4 during hESC differentiation. Related to the introduction and Fig. 4. Figure S2. Effects of SOX2 expression in hESCs. Related to Fig. 1 and Fig. 2. Figure S3. SOX2-OE hESCs cultured in KSR medium. Related to Fig. 3. Figure S4. OCT4 and PAX6 co-express transiently in early neural differentiation and bind to the same SOX2 HMG domain motif. Related to Fig. 4. Figure S5. Analysis of SOX2-OCT4 and SOX2-PAX6 ChIP-Seq datasets. Related to Fig. 5. Figure S6. OCT4 and BRN2 enhancers. Related to Fig. 6. Table S1. Primers used in the study. Related to Figs. 1, 2, 3 and 6 and Figures S1, S3-S4. Table S2. Antibodies used in the study. Related to Figs. 1, 2, 3, 4 and 6 and Figure S1-S2 and S4. (PDF 1426 kb) [file 13287_2019_1228_MOESM1_ESM.pdf]

Supplemental Figure and Figure Legends

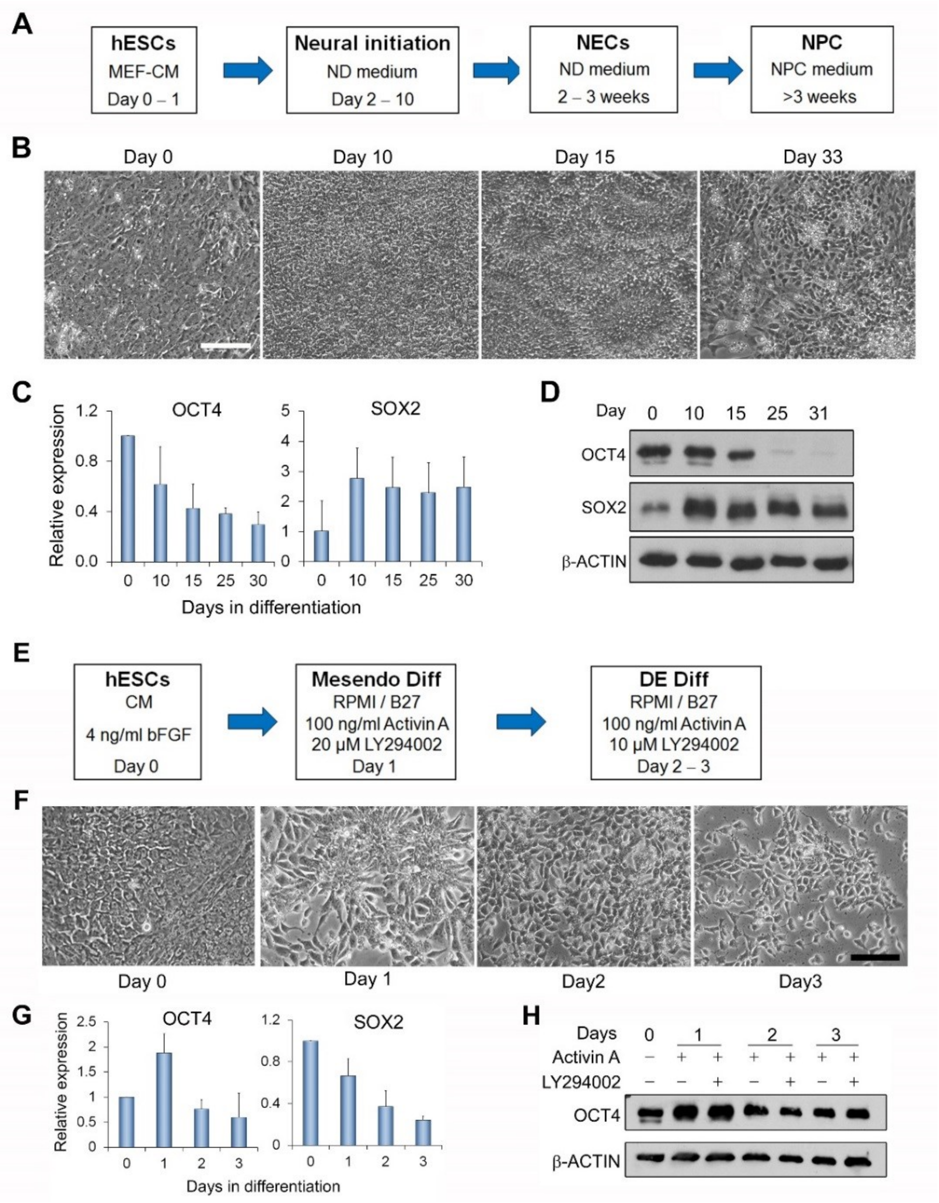

Fig. S1. Distinct dynamic expression of SOX2 and OCT4 during hESC differentiation. Related to introduction and Fig. 4. (A) Schematic diagram depicting hESC neural differentiation. MEF-CM, mouse embryonic fibroblast-conditioned medium; ND, neural differentiation; NEC: neural epithelial cell; NPC, neural progenitor cell. (B) Representative cell images during hESC neural differentiation. The days in differentiation are indicated. Scale bar = 100 μm. (C) OCT4 and SOX2 mRNA levels during neural differentiation by qRT-PCR. Data are presented as fold changes relative to day 0 of the hESCs. Error bars represent mean ± SD of 6-9 analysed samples from two independent differentiations. (D) Immunoblot showing OCT4 and SOX2 protein levels as in C. (E) Illustrative diagram depicting hESC DE differentiation. (F) Representative cell images during hESC DE differentiation. The days in differentiation are indicated. Scale bar = 100 μm. (G) OCT4 and SOX2 mRNA levels during DE differentiation by qRT-PCR. Data presented as in C. (H) Immunoblotting showing OCT4 and SOX2 protein levels during DE differentiation of hESCs.

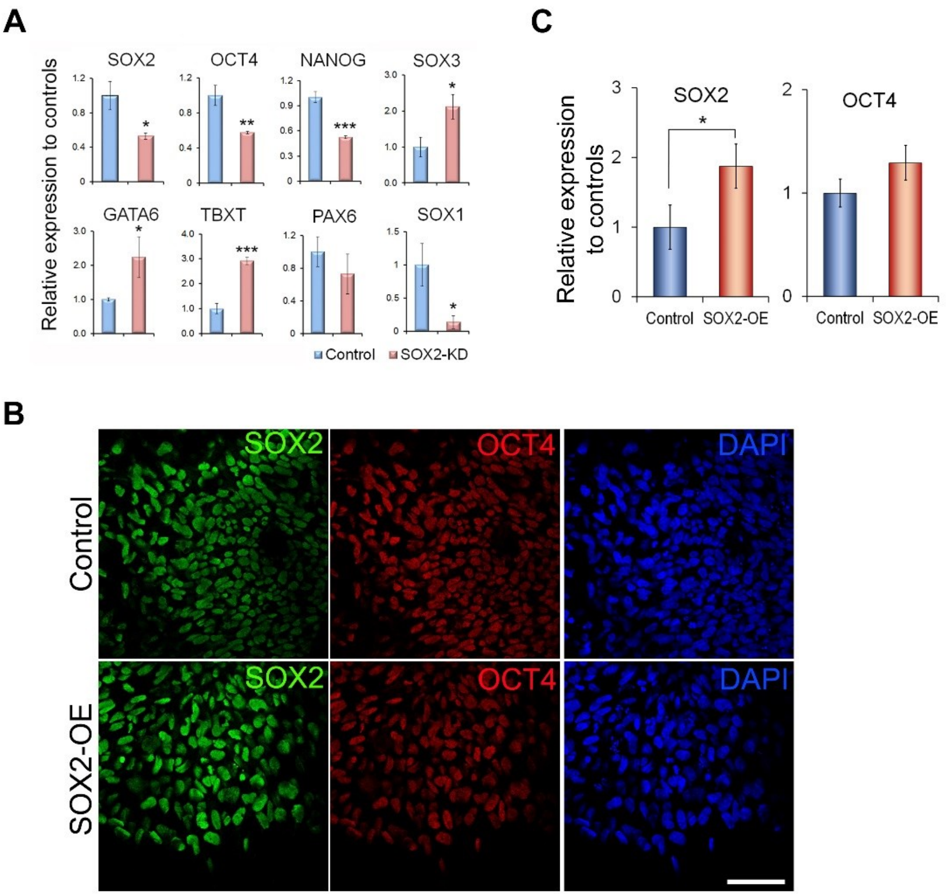

Fig. S2. Effects of SOX2 expression in hESCs. Related to Fig. 1 and Fig. 2. (A) The indicated mRNA expression in H7 hESCs 4 days after they were infected with lentivirus carrying SOX2 (SOX2-KD) or scrambled (Control) shRNAs as in Fig. 1C. (B) Immunostaining of control and SOX2-overexpressing (SOX2-OE) H1 hESCs with OCT4 and SOX2 antibodies. (C) SOX2 and OCT4 mRNA expression in FACS-sorted Tra-1-81+ SOX2-OE and control H1 hESCs. Data are presented as mean ± SD from 3 independent SOX2-KD or SOX2-OE cell lines with triplicate PCR for each sample.

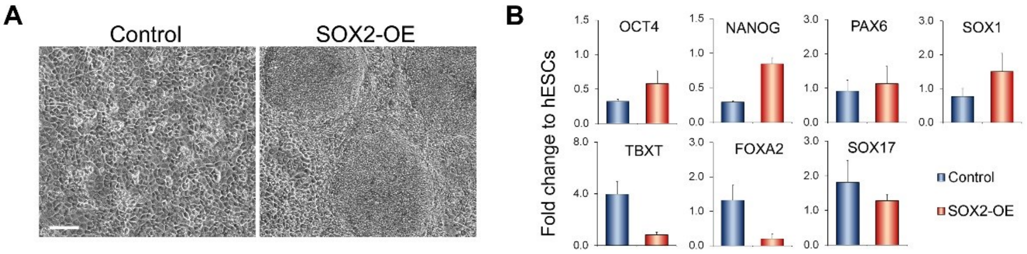

Fig. S3. SOX2-OE hESCs cultured in KSR medium. Related to Fig. 3. (A) Representative cell images of SOX2-OE and control cells in KSR medium for ~30 days. Scale bar = 100 μm. **(B)** mRNA expression of indicated markers by qRT-PCR. Error bars represent mean $\pm$ SD of 6-9 analysed samples from two independent differentiations.

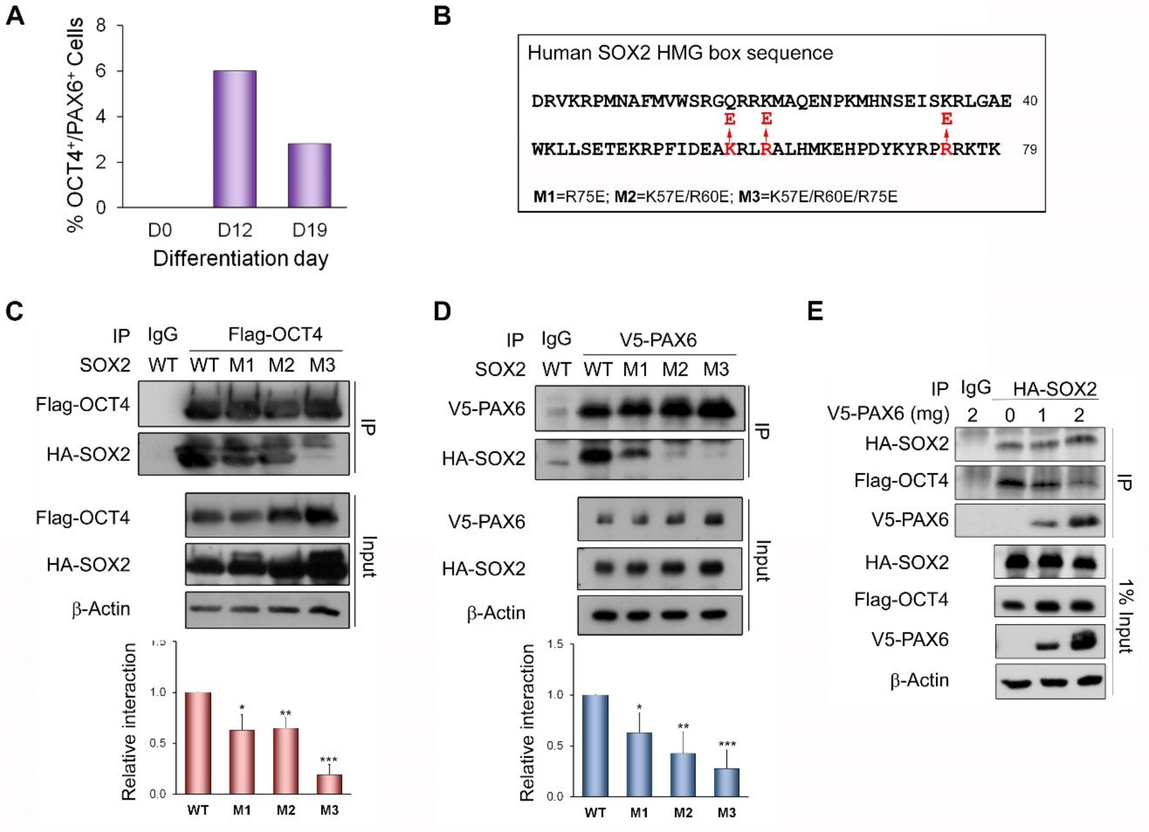

Fig. S4. OCT4 and PAX6 co-express transiently in early neural differentiation and bind to the same SOX2 HMG domain motif. Related to Fig. 4. (A) Percentage of cells expressing both PAX6 and OCT4 at the indicated days during neural differentiation analyzed by single cell RNA-seq (Yao et al., 2017). (B) Illustration of SOX2 HMG box sequence with the mutated residues indicated. (C) Co-IP showing Oct4 interactions with the indicated Sox2 mutants in (B) after co-transfection into HEK293T cells. *, **, *** represent p < 0.05, 0.01, 0.005 by student *t* test relative to WT (n=3). (D) Co-IP experiments performed similar to (C) showing Pax6 interactions with the same SOX2 mutants. (E) Co-transfection of HA-SOX2 and Flag-OCT4 into HEK293T cells with increasing dosages of V5-PAX6 as indicated. Then the cell lysates were isolated and immunoprecipitated with HA-SOX2 antibody, and the immunoprecipitates were immunoblotted with Flag-OCT4 and V5-PAX6 antibodies.

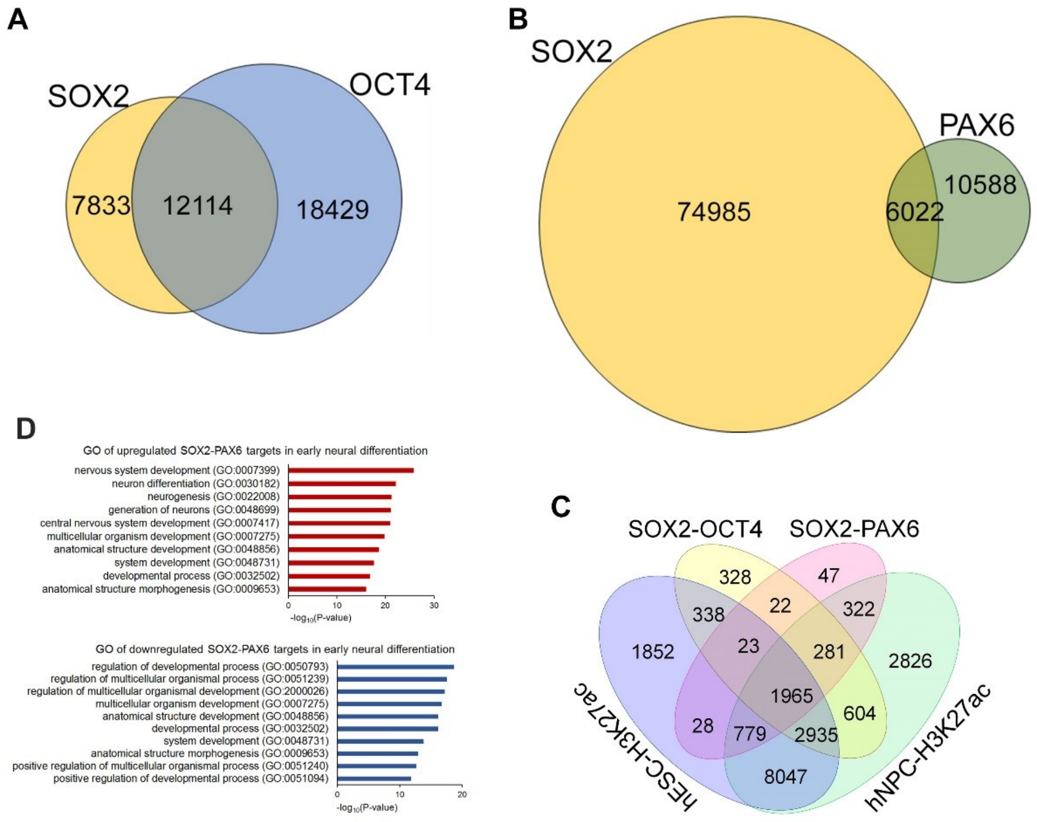

Fig. S5. Analysis of SOX2-OCT4 and SOX2-PAX6 ChIP-seq datasets. Related to Fig. 5. (A) Venn diagram of SOX2 and OCT4 ChIP-Seq peak overlapping in hESCs. (B) Venn diagram of SOX2 and PAX6 ChIP-Seq peak overlapping in hNPCs. (C) Venn diagram of analysis summary of SOX2-OCt4 and SOX2-PAX6 target genes in relation to H3K27ac in hESCs and hNPCs. (D) GO analysis of biological process of upregulated (≥1.5 fold of hESC level) and downregulated (≤0.5 fold of hESC level) SOX2-PAX6 target genes in the early neural differentiating cells.

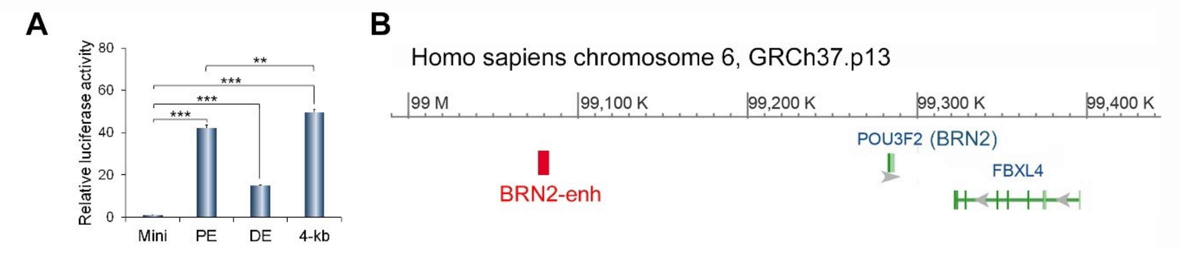

Fig. S6. OCT4 and BRN2 enhancers. Related to Fig. 6. (A) OCT4-luciferase activities in hESCs 48 hours after the transfection. Data are presented as mean ± SD of three independent transfection experiments. *** and ** represent $p<0.0001$ and 0.005, respectively, by student t test. Mini represents minimal promoter, PE and DE represent proximal and distal enhancers. (B) Schematic map of human BRN2 (*POU3F2*) locus. Identified enhancer region, which is bound by SOX2 and PAX6, is marked in red.

Supplemental Tables

Table S1: Primers used in the study, related to Figs. 1-3 and 6, Figs. S1, S3-S4.

**For plasmid cloning**	**Forward (5' - 3')**	**Reverse (5' - 3')**
SOX2 cDNA FseI/AscI	TATAGGCCGGCCAGATCTTATGT	AATTGGCGCGCCACTAGTTCACATGTGT
Sox2-mutant-R75E	ATACCGGCCCGAGCGGAAAACCA	ATCCGGGTGCTCC
Sox2-mutant-K57E, R60E	CTGGAAGAGCGCTGCACATGAAGG	CCGTTCAGCCTCGTCGATGAACGG
OCT4 cDNA	TTAAACTAGTATGGCGGGACACCTG	TATAGGATCCTTAGTTTGAATGCATGGGAGA
PAX6 cDNA	ATATGGATCCTATTCGAGCCCCGTGGAATC	GCGACTCGAGTGTAATCTTGGCCAGTATTG
BRN2 enhancer	AATTGGTACCACTGCATAAATGGTCTAGTGGG	AATTGCTAGCTCGGAATGGATCACAGATTTTTC

**For ChIP-qPCR**	**Forward (5' - 3')**	**Reverse (5' - 3')**
Oct4-PE	ACCAGGCCCCATAATCTACC	TTCCCCCACTCTTATGTTGC
Oct4-DE	TGAGAAACACTGGTGTGGAGAT	TCTCAATCCCCAGGACAGAA
GAPDH 2nd intron	AATGAATGGGCAGCCGTTAG	AGCTAGCCTCGCTCCACCTGAC

**For qPCR**	**Forward (5' - 3')**	**Reverse (5' - 3')**
β-ACTIN	AGAGCTACGAGCTGCCTGAC	AGCACTGTGTTGGCGTACAG
ASCL1	GGAGCTTCTCGACTTCACCA	CTAAAGATGCAGGTTGTGCG
Brachyury (TBXT)	TGCTTCCCTGAGACCCAGTT	GATCACTTCTTTCCTTTGCATCAAG
BRN2	CCTCGTAAGGGGAATGTG	ATCGGAGAGAGTTGAAGCCA
CDX2	GGATGGTGATGTAGCGACTGT	ACCTGTGCGAGTGGATGC
EOMES	AGGAATTCTTGCTTTGCTAATTCTG	CGAAGAAACAGCAAGAGCAGC
GATA6	ACTTGAGCTCGCTGTTCTCG	CAGCAAAAATACTTCCCCCA
FOXA2	GGGAGCGGTGAAGATGGA	TCATGTTGCTCACGGAGGAGTA
GFAP	CACCACGATGTTCCTCTTGA	GTGCAGACCTTCTCCAACCT
NESTIN	GAGGGAAGTCTTGGAGCCAC	AAGATGTCCCTCAGCCTGG
NANOG	TGATTTGTGGGCCTGAAGAAAA	GAGGCATCTCAGCAGAAGACA
OCT4	TCGAGAACCGAGTGAGAGGC	CACACTCGGACCACATCCTTC
PAX6	TCCGTTGGAACTGATGGAGT	GTTGGTATCCGGGGACTTC
RPL22	TCGCTCACCTCCCTTTCTAA	TCACGGTGATCTTGCTCTTG
SOX1	AACACTTGAAGCCCAGATGGA	GCAGGCTGAATTCGGTTCTC
SOX17	ACGCCGAGCTCAGCAAGAT	TCCACGTACGGCCTCTTCTG
SOX2	GCCGAGTGGAAACTTTTGTCG	GCAGCGTGTACTTATCCTTCTT
SOX21	CCACTCGCTTGGATTTCTGACACA	TCGACTCAAACTTAGGGCAACGA
SOX3	TGGAGAACTGCAACGCCTACGC	GATCACGGCAGAAATCACCAACTC

Table S2: Antibodies used in the study, related to Figs 1-4, 6 and Figs S1-S2 and S4.

Antibodies	Campany: Cat#	Dilutions
Brachyury	R&D: AF2085	IB: 1:500
Sox17	R&D: MAB1924	IB: 1:500
GATA6	Abcam: ab22600	IB: 1:400
Flag	Sigma: F1804	IB: 1:1000, IP: 1:100
Tuj1 (β-tubulin III)	Sigma-T8660	IF:1:1000
FoxA2	Abcam: ab60721	IB: 1:200
Caspase 3	Cell Signalling: 9662	IF:1:200
HA	Sigma: H3663	IB:1:1000 IF :1:100
Nestin	R&D: MAB1259	IF: 1:100
Oct-04	Santa cruz: sc-5279	IB 1:400 IF 1:100
Pax6	Millipore: AB2237	IB：1:1000 IF：1:200
Pax6	Abcam: 109233	IB: 1:1000
Phospho Histone H3 (Ser10)	Millipore: 05-806	IF 1:200
Sox2	R&D : AF2018	IB: 1:400 IF: 1:40
Tra-1-81	Santa Cruz: sc-21706	IF: 1:50
β-actin	Sigma: A5316	IB: 1:5000
α-tubulin	Cell Signaling:10376	IB: 1:200
MC-480 (SSEA-1)	DSHB: MC480	IF: 1:5
Goat IgG-HRP	Santa Cruz: 2020	IB: 1:5000
Mouse IgG-HRP	Santa Cruz: 2005	IB: 1:5000
Mouse IgG-HRP light chain	Stratech: 211-032-174	IB: 1:10000
Normal mouse IgG2a	Santa Cruz: 2025	IP: µg equivalent to HA
Normal rabbit IgG	Santa Cruz: 2027	IP: µg equivalent to SOX2
Rabbit IgG-Alexa Fluor 488	Life Technologies: A11055	IF: 1:400
Rabbit IgG-Alexa Fluor 568	Life Technologies A11011	IF:1:400
Rabbit IgG-HRP	Santa Cruz: 2004	IB: 1:2000
Rabbit IgG-HRP light chain	Stratech: 211-032-171	IB: 1:10000
Mouse IgG-Alexa Fluor 488	Life Technologies: A11001	IF 1:400
Mouse IgG Alexa Fluor 568	Life Technologies: A11004	IF 1:400
